# Expression of Phosphorylated BRD4 Is Markedly Associated with the Activation Status of the PP2A Pathway and Shows a Strong Prognostic Value in Triple Negative Breast Cancer Patients

**DOI:** 10.3390/cancers13061246

**Published:** 2021-03-12

**Authors:** Marta Sanz-Álvarez, Ion Cristóbal, Melani Luque, Andrea Santos, Sandra Zazo, Juan Madoz-Gúrpide, Cristina Caramés, Cheng-Ming Chiang, Jesús García-Foncillas, Pilar Eroles, Joan Albanell, Federico Rojo

**Affiliations:** 1 Pathology Department, Fundación Jiménez Díaz University Hospital Health Research Institute (IIS—FJD, UAM)-CIBERONC, E-28040 Madrid, Spain; marta.sanza@quironsalud.es (M.S.-Á.); melani.luque@quironsalud.es (M.L.); szazo@fjd.es (S.Z.); JMadoz@fjd.es (J.M.-G.); 2 Cancer Unit for Research on Novel Therapeutic Targets, Oncohealth Institute, IIS-Fundación Jiménez Díaz-UAM, E-28040 Madrid, Spain; andrea.santos@quironsalud.es (A.S.); ccarames@fjd.es (C.C.); 3 Departments of Biochemistry and Pharmacology, Simmons Comprehensive Cancer Center, University of Texas Southwestern Medical Center, Dallas, TX 75390, USA; chen-ming.chiang@UTSouthwestern.edu; 4 Medical Oncology Department, University Hospital “Fundación Jiménez Díaz”, UAM, E-28040 Madrid, Spain; jesus.garciafoncillas@oncohealth.eu; 5 Institute of Health Research INCLIVA-CIBERONC, E-46010 Valencia, Spain; pilar.eroles@uv.es; 6 Medical Oncology Department, Hospital del Mar-CIBERONC, E-08003 Barcelona, Spain; 96087@parcdesalutmar.cat

**Keywords:** pBRD4, SET, PP2A, prognosis, triple negative breast cancer

## Abstract

**Simple Summary:**

The use of BRD4 inhibitors has emerged as a novel therapeutic approach in a wide variety of tumors including the triple negative breast cancer. Moreover, PP2A has been proposed as the phosphatase involved in regulating BRD4 phosphorylation and stabilization. Our aim was to evaluate for the first time the clinical impact of BRD4 phosphorylation in triple negative breast cancer patients and as well as its potential linking with the PP2A activation status in this disease. Our findings are special relevant since they suggest the prognostic value of BRD4 phosphorylation levels, and the potential clinical usefulness of PP2A inhibition markers to anticipate response to BRD4 inhibitors.

**Abstract:**

The bromodomain-containing protein 4 (BRD4), a member of the bromodomain and extra-terminal domain (BET) protein family, has emerged in the last years as a promising molecular target in many tumors including breast cancer. The triple negative breast cancer (TNBC) represents the molecular subtype with the worst prognosis and a current therapeutic challenge, and TNBC cells have been reported to show a preferential sensitivity to BET inhibitors. Interestingly, BRD4 phosphorylation (pBRD4) was found as an alteration that confers resistance to BET inhibition and PP2A proposed as the phosphatase responsible to regulate pBRD4 levels. However, the potential clinical significance of pBRD4, as well as its potential correlation with the PP2A pathway in TNBC, remains to be investigated. Here, we evaluated the expression levels of pBRD4 in a series of 132 TNBC patients. We found high pBRD4 levels in 34.1% of cases (45/132), and this alteration was found to be associated with the development of patient recurrences (*p* = 0.007). Interestingly, BRD4 hyperphosphorylation predicted significantly shorter overall (*p* < 0.001) and event-free survival (*p* < 0.001). Moreover, multivariate analyses were performed to confirm its independent prognostic impact in our cohort. In conclusion, our findings show that BRD4 hyperphosphorylation is an alteration associated with PP2A inhibition that defines a subgroup of TNBC patients with unfavorable prognosis, suggesting the potential clinical and therapeutic usefulness of the PP2A/BRD4 axis as a novel molecular target to overcome resistance to treatments based on BRD4 inhibition.

## 1. Introduction

Breast cancer has the highest prevalence in cancer diagnosis and represents the second leading cause of female cancer-related deaths [1]. Breast cancer is a very heterogeneous disease, with different molecular subtypes including luminal A, luminal B, HER2+, basal and normal-like tumors [2,3]. The triple negative breast cancer (TNBC) is molecularly characterized by the lack of hormonal receptors expression (estrogen (ER) and progesterone receptors (PR)), and by an absence of expression of the HER2 receptor [3]. TNBC represents 15–20% of all breast carcinomas [4] and shows more aggressive features such as emergence at a younger age, higher tumor size and grade, and greater proportion of positive lymph node metastases. TNBC has been largely described as the breast cancer subtype with the worst overall and progression-free survival rates, and represents a major challenge for current clinical management due to the lack of established and effective therapeutic strategies [5,6]. TNBC cells have very aggressive behavior that leads to a shorter time of disease progression. In fact, TNBCs show the highest recurrence rates, with brain and visceral organs as the main metastatic niches [7]. Triple negative tumors are heterogeneous at the molecular level, and *TP53*, *PIK3CA*, *PTEN*, *RB1*, *EGFR* and *MYC* have been reported as the most commonly mutated genes [8,9]. However, it remains urgent to improve our understanding about the molecular alterations that govern TNBC progression in order to develop novel therapeutic strategies for this disease.

Bromodomain-containing protein 4 (BRD4) is a member of the bromodomain and extra-terminal domain (BET) protein family, along with BRD2, BRD3, and BRDT. BRD4 is structurally composed of two N-terminal bromodomain domains (BD1 and BD2), and a C-terminal extra-terminal domain. BD1 and BD2 allow for the formation of a hydrophobic pocket that binds to acetylated lysine residues of histones or transcription factors [10,11], ultimately regulating a wide variety of cell functions. Specifically, BRD4 is involved in chromatin decompaction, the recruitment of components of the transcriptional complex, as well as in the stages of initiation, release pause and elongation of transcription due to its interaction with PTEF-b that phosphorylates RNA Pol II [10]. Due to its role in important cellular processes, BRD4 dysfunction can lead to the appearance of various human diseases, including inflammation, cardiovascular diseases and cancer [10,11,12]. BRD4 has been found to play oncogenic roles in many hematological and solid tumors, including melanoma, prostate and breast cancer among others, and has been proposed as a druggable promising target in human cancer [12,13,14,15,16]. BRD4 has been shown to regulate the expression of different set of oncogenic drivers, such as c-MYC [13], NF-κB [16] or Jagged1 [17]. In breast cancer, several BRD4 alterations involved in the different molecular subtypes have been reported to date. Thus, BRD4 activity has been found to be required for proliferation and ERα function in ER+ breast cancer cells [18], and promotes the migration and invasion of triple negative tumors through controlling Jagged1 expression [17]. Regarding its post-translational modifications, CK2-mediated BRD4 hyperphosphorylation has been associated with greater stability and nuclear localization of the BRD4 protein [19], with important functional and therapeutic implications in TNBC [20,21]. In fact, the therapeutic value of BRD4 inhibition in TNBC has been previously reported by Shu and co-workers [21], analyzing a set of BRD4 inhibitors across a panel of cell lines with different breast cancer subtypes, observing that these drugs showed the strongest antitumor effects in the triple negative subtype. These results were confirmed in vivo using primary human TNBC xenografts. After an exhaustive analysis of potential mechanisms of drug resistance, BRD4 was identified as a novel PP2A target and its hyperphosphorylation as an alteration that promotes resistance to BRD4 inhibition in TNBC cells.

In the last years, several studies have evaluated distinct therapeutic approaches related totargeting BRD4 in TNBC. It has been reported promising antitumor properties using cell-penetrating peptides including EGFR and BRD4 siRNAs in TNBC cells [22], or a dual-target small-molecule inhibitor co-targeting PARP1 and BRD4 [23]. Moreover, it has been described that BRD4 regulates PD-L1 expression in TNBC cells, which could have interesting implication for immunotherapy-based treatments [24], or the therapeutic usefulness of strategies based on BRD4 inhibition, due to its role as regulator of the oncogenic c-MYC pathway in this disease [25,26].

Altogether, the different studies in the literature regarding BRD4 in TNBC highlight its promising therapeutic value. However, little is known about its clinical impact as well as the functional and therapeutic significance of pBRD4 in this disease. Moreover, the relevance of the PP2A pathway as a potential regulator of pBRD4 remains to be investigated and confirmed in TNBC patient cohorts.

## 2. Experimental Section

### 2.1. Patient Samples

A total number of 132 surgical resection specimens from patients diagnosed withprimary breast cancer were included in this study. Formalin-fixed paraffin-embedded breast tumor specimens from this patient cohort were retrospectively selected from Fundación Jiménez Díaz Biobank (Madrid, Spain) following these criteria: infiltrating carcinomas, operable, enough available tissue, molecular and/or clinical follow-up data and triple negative subtype. Clinical data were collected from medical clinical records by oncologists. Samples were taken anonymously. TNM (tumor–node–metastasis) staging classification was performed using the American Joint Committee on Cancer (AJCC) staging system. The Scarff–Bloom–Richardson modified by Elston criteria [27] was used to define the histological grade. Two independent pathologists who were blinded to patient outcomes evaluated tumor tissue staining.

### 2.2. Determination of the Molecular Subtype

We evaluated the expression of hormonal receptors as well as HER2 to define the molecular subtype and confirm that all patients included in this study have triple negative breast tumors. The expression of both estrogen receptor (ER) and progesterone receptor (PR) were determined by immunohistochemistry (IHC) (SP1 and PgR636 clones, respectively; Dako, Carpinteria, CA, USA), establishing positivity criteria in >1% of nuclear tumor staining [28]. Determination of HER2 amplification was carried out by FISH (Pathvysion; Abbott Laboratories, Green Oaks, IL, USA) [29].

### 2.3. Ethics Approval and Consent to Participate

This study was conducted in full accordance with the guidelines for Good Clinical Practice and the Declaration of Helsinki. All participants gave written informed consent for tissue storage and analysis at Fundación Jiménez Díaz biobank, Madrid (Spain). The ethical committee institutional review board of Fundación Jiménez Díaz University Hospital reviewed and approved the project (ref. PIC 13-2016).

### 2.4. Immunohistochemistry

Representative areas of each tumor were carefully selected, and three tissue cores (1mm diameter) were obtained using a tissue microarray (TMA) workstation (T1000 Chemicon). Immunostainings were performed on tissue sections (3 μM) obtained from FFPE tumors, as previously described [30]. Expression levels of Ki-67 were studied by IHC using the MIB1 clone (Dako, Carpinteria, CA, USA) [31]. High proliferation in our breast cancer patient cohort based on Ki-67 labelling by IHC has been defined following the 13th St Gallen International Breast Cancer Conference (2013) criteria based on a threshold ≥ 20% of proliferation [32]. Other antibodies used were: pBRD4 (developed and kindly provided by Prof. Chiang’s laboratory) [19,21], rabbit polyclonal anti-SET (ab1183) (Abcam, Cambridge, UK) and rabbit monoclonal anti-PP2AY307 (1155-1) (Abcam, Cambridge, UK). Antibody dilutions were as follows: pBRD4 (1:100), SET (1:5000), and phospho-PPP2CA (pPPP2CA) (1:2000). pBRD4, SET and pPPP2CAexpression blinded to clinical data was evaluated by two pathologists (F.R. and S.Z.). The specific phosphorylation sites recognized by the antibodies were Y307 for PPP2CA and S484/488 for BRD4. A semiquantitative histoscore (Hscore) was calculated by estimation of the percentage of tumor cells positively stained with low, medium, or high staining intensity. The final score was determined after applying a weighting factor to each estimate. The formula used was Hscore = (low%) × 1 + (medium%) × 2 + (high%) × 3, and the results ranged from 0 to 300.

### 2.5. Statistical Analysis

Statistical analyses were performed using SPSS20 for windows (SPSS Inc, Chicago IL, USA). We applied the χ^2^ test (Fisher exact test) based on bimodal distribution of data to evaluate the significance of potential associations between BRD4 phosphorylation and the molecular and clinical characteristics of the tumor specimens included in this study.

Overall survival (OS) was defined as the time from diagnosis to the date of death from any cause or last follow-up. Event-free survival(EFS)was defined as the time from the date of diagnosis until relapse at any location, death or last follow-up. Kaplan–Meier plots and survival comparisons were carried out using the log-rank test if the proportional hazard assumption was fulfilled, and Breslow otherwise. The Cox proportional hazards model was adjusted taking into consideration significant parameters in the univariate analysis. A receiver operating characteristic (ROC) curve was used to determine the optimal cutoff point based on progression end point for pBRD4 as previously described [33,34]. *p*-Value less than 0.05 was considered statistically significant. This work was carried out in accordance with Reporting Recommendations for Tumor Marker Prognostic Studies(REMARK) guidelines [35].

## 3. Results

### 3.1. Prevalence of BRD4 Hyperphosphorylation in Triple Negative Breast Cancer Patients and Its Association with Molecular and Clinical Parameters

To investigate the prevalence and potential clinical impact of pBRD4 in TNBC, we analyzed the expression of pBRD4 by immunohistochemistry in a cohort of 132 patients with early breast cancer and triple negative subtype, observing high pBRD4 levels in 45 of 132 of cases (34.1%). Patient characteristics are presented in Table S1. We next correlated pBRD4 expression with molecular and clinical features of our patient cohort. Interestingly, high pBRD4 levels were found to be strongly associated with the subgroup of patients who relapsed (*p* = 0.007). Associations between pBRD4 status and clinical and molecular characteristics are shown in Table 1.

### 3.2. Clinical Impact of pBRD4 in Triple Negative Breast Cancer

**Figure 1:**
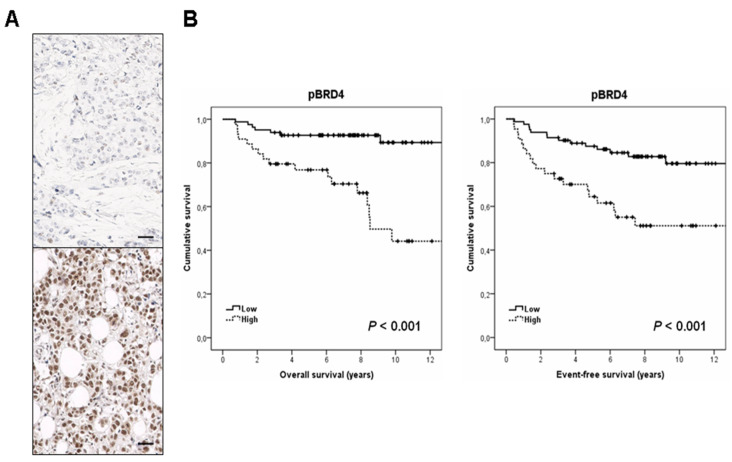
Clinical significance of pBRD4 in TNBC. (**A**) Immunohistochemical images showing pBRD4 positive and negative staining. The line shows 25 µm. Original magnification ×400, (**B**) Kaplan–Meieranalysesof overall survival(OS) and event-free survival(EFS) in a cohort of 132 TNBC patients.

We analyzed the clinical significance of pBRD4 in the same cohort of 132 TNBC patients. Clinical follow-up data were available in all cases. The median of age was 57 years (with an age range of 31 to 90 years). Interestingly, we found that the subgroup of high pBRD4 expressing patients had a markedly shorter OS (*p* < 0.001) (Figure 1A). Moreover, we observed that pBRD4 also had predictive value for EFS in our patient cohort (*p* < 0.001) (Figure 1B).

Interestingly, multivariate Cox analysis showed that high pBRD4 expression is an unfavorable independent factor associated with patient outcome in our cohort (Hazard ratio (HR) = 5.342; 95% confidence interval (CI), 2.286–12.482; *p* < 0.001) (Table 2).

To further evaluate the prognostic value of pBRD4 in TNBC, we stratified our patient cohort by stage. Of note, we observed that relevance of high pBRD4 expression levels as a biomarker predictor of poor outcome was retained in all cases for both OS and EFS, but the significance was particularly marked in the subgroup of TNBC patients with stage III (*p* < 0.001 for OS, and *p* = 0.001 for EFS), compared to those with stages I-II (*p* = 0.005 for OS, and *p* = 0.017 for EFS) (Supplementary Materials Figure S1).

### 3.3. BRD4 Phosphorylation Is Associated with the Activation Status of the PP2A Pathway

We next studied the molecular mechanisms that could be involved in BRD4 hyperphosphorylation. Due to BRD4 having been previously proposed as a direct target of the tumor suppressor protein phosphatase 2A (PP2A) in TNBC, the activation status of this phosphatase was evaluated in our patient series. The phosphorylation of the PP2A catalytic subunit in its tyrosine 307, as well as the overexpression of endogenous inhibitors such as SET, have been reported as major contributing alterations to inhibit PP2A in human cancer. Thus, we analyzed both pPPP2CAand SET levels in 128 TNBC cases from our cohort with enough material available. High pPPP2CAexpression was found in 31 out of 128 cases (24.2%), whereas 17 out of 128 cases (13.3%) showed SET overexpression. Interestingly, we found that high pBRD4 expression was strongly associated with both PP2A hyperphosphorylation (*p* < 0.001) and SET overexpression (*p* < 0.001) (Table 3), which highlights that PP2A inhibition could be a key molecular mechanism to maintain BRD4 phosphorylation in TNBC.

Since pPPP2CA and SET have been described to be associated alterations, we analyzed how many patients had a concomitant PP2A hyperphosphorylation and SET overexpression. As expected, we observed a significant correlation between both alterations (*p* < 0.001), which were found in 12 cases from our series (Table S2). Moreover, we also analyzed the prognostic value of pPPP2CA in our patient cohort. As expected, those patients with high pPPP2CA expression levels showed a significantly worse OS (*p* < 0.001) and EFS (*p* < 0.001) (Figure S2).

## 4. Discussion

The TNBC subtype has been previously reported to be particularly sensitive to the treatment with bromodomain inhibitors. In addition, BRD4 hyperphosphorylation has been defined as a molecular alteration that promotes resistance to BRD4 inhibitors, and the tumor suppressor PP2A as the major regulator of BRD4 dephosphorylation. However, the potential clinical impact of this pBRD4 together with the validation of its linking with the PP2A activation status remain to be fully clarified in TNBC patients. It has been recently reported that BRD4 expression is significantly higher in breast cancer tissues than in normal controls, and defines poor prognosis in breast cancer patients [36]. These results would further strengthen our findings in the present study, especially considering that BRD4 phosphorylation has been described as an alteration involved in BRD4 protein stabilization [21]. Moreover, we observed that the prognostic impact of pBRD4 was particularly evident in stage III TNBC patients (Figure S1). This observation, together with the fact that this alteration is associated with recurrence (Table 1), would suggest that BRD4 hyperphosphorylation could be an event with functional relevance in TNBC progression. Thus, its evaluation in a TNBC cohort with metastatic disease would be of high interest in forthcoming studies.

The fact that decreased PP2A activity has been described to induce in vitro BRD4 hyperphosphorylation and resistance to BRD4 inhibition [21] prompted us to analyze the PP2A activation status in our patient cohort. PP2A is a key tumor suppressor commonly deregulated in human cancer [37]. PP2A hyperphosphorylation, as well as upregulation of the endogenous PP2A inhibitors such as SET, has been reported as main molecular mechanisms of PP2A inhibition in many tumors including breast cancer. These alterations have progressively emerged as promising therapeutic targets in this disease [38,39,40,41,42,43,44]. Although it has been recently reported that PP2A inhibition is a frequent alteration in breast cancer related with poor outcome and therapy resistance, such studies have been carried out in cohorts including cases with different molecular subtypes [40,45,46]. Therefore, the evaluation of the precise PP2A status in a cohort of TNBC patients as well as its clinical impact in this breast cancer subtype remains still to be performed. Previous works have shown that the PP2A inhibitor CIP2A confers poor outcome in TNBC cells, which has been recently confirmed in the work by Tawab Osman and co-workers [47,48,49]. These findings would suggest that PP2A inhibition could be of relevance in this breast cancer subtype. In fact, we found in this work that high pPPP2CA were predictor of poor outcome in our TNBC patient cohort (Figure S2). We observed PP2A hyperphosphorylation in 24.2% of cases (31/128) and SET overexpression in 13.3% of cases (17/128). Both alterations were present in 12 patients from our cohort, indicating that 5 patients had SET overexpression without high pPPP2CAexpression, and 19 cases only showed high pPPP2CAlevels. Thus, 82.9% of cases (34/41) with BRD4 hyperphosphorylated had at least one of the PP2A inhibitory markers altered. Therefore, our results suggest that both PP2A hyperphosphorylation and SET overexpression could be molecular contributing alterations to enhance BRD4 phosphorylation levels in TNBC, but it remains to be experimentally confirmed. Only 2 out of 31 cases with high pPPP2CA had low pBRD4 expression. However, the observation that 7 pBRD4 overexpressing patients without any PP2A inhibitory alteration detected would also indicate the potential existence of alternative PP2A inhibitory alterations or molecular mechanisms distinct that PP2A inhibition that deregulate pBRD4 in this disease. Altogether, these results are in concordance with the conclusions reported by Shu and co-workers [21] identifying PP2A as the phosphatase responsible of dephosphorylating BRD4. However, they did not observe prognostic value for pBRD4 and discrepancies in clinical impact may be due to sample size and the fact that those authors stratified their cohort by pBRD4 expression using a median split of pBRD4 intensity.

Furthermore, these findings are of therapeutic relevance, since the use of PP2A activators could serve to overcome a foreseeable development of resistance to BRD4 inhibitors in TNBC patients with high pBRD4 levels. In fact, Shu and co-workers showed that the combination of the PP2A activator perphenazine with JQ1 served to overcome resistance to BRD4 inhibitors in TNBC cells [21]. In this line of thinking, FTY720 is an FDA-approved immunosuppressant used to treat multiple sclerosis, which has shown potent antitumor effects in many tumor types [50]. Moreover, FTY720 has been described as a PP2A activating drug through targeting pPPP2CA and SET, which are the PP2A inhibitory alterations reported in this work. Another relevant issue is the fact that BRD4 is expressed in two major isoforms, short and long, that have been reported to play opposite functions as regulators of gene transcription and tumor progression [51]. The antibody used in our work recognizes phosphorylation on S484/488, which is a region present in both BRD4 isoforms. Therefore, we analyzed here by IHC the total levels of pBRD4 expression, corresponding to the contribution of the long and short BRD4 isoforms. However, it would be of high interest to investigate the potential functional and clinical implications derived from the phosphorylation of each BRD4 isoform separately. Altogether, our results show that high pBRD4 levels define a subgroup of TNBC cases with very poor outcomes. Moreover, our findings are consistent with PP2A inhibition as a key molecular mechanism to induce BRD4 hyperphosphorylation in TNBC patients, which could benefit from a future inclusion of PP2A activators and BRD4 inhibitors in clinical protocols. Moreover, it would be of high interest to study the potential benefit derived from the clinical use of PP2A activators to anticipate and overcome the development of resistance to BRD4 inhibition in TNBC.

## 5. Conclusions

In conclusion, BRD4 hyperphosphorylation is a frequent alteration that associates with patient recurrence and independently predicts shorter OS and EFS in TNBC patients. Moreover, we observe a molecular background based on PP2A inhibition as the potential molecular mechanism that contributes to enhanced pBRD4 levels. Altogether, our findings highlight the clinical impact of pBRD4, as well as the PP2A/pBRD4 signaling axis as a novel therapeutic target in TNBC, which needs to be fully confirmed in forthcoming studies.

## Figures and Tables

**Table 1:** Association of bromodomain-containing protein 4 (BRD4) phosphorylation levels with molecular and clinical parameters in a cohort of 132 triple negative breast cancer (TNBC) patients.

Parameters	No. Cases	No. Low pBRD4 (%)			No. High pBRD4 (%)		*p*
pBRD4	132	87	(65.9)		45	(34.1)	
Hormonal status	132	87			45		0.261
Premenopausal	36	21		(24.1)	15	(33.3)	
Postmenopausal	96	66		(75.9)	30	(66.7)	
Morphological type	131	86			45		0.427
IDC ^1^	122	79		(91.9)	43	(95.6)	
ILC ^2^	9	7		(8.1)	2	(4.4)	
T ^3^	132	87			45		0.377
1	54	32		(36.8)	22	(48.9)	
2	60	43		(49.4)	17	(37.8)	
3–4	18	12		(13.8)	6	(13.3)	
N ^4^	132	87			45		0.457
0	77	52		(59.8)	25	(55.6)	
1	33	19		(21.8)	14	(31.1)	
2–3	22	16		(18.4)	6	(13.3)	
Stage	132	87			45		0.865
I	39	25		(28.7)	14	(31.1)	
II	60	41		(47.1)	19	(42.2)	
III	33	21		(24.2)	12	(27.7)	
Grade	132	87			45		0.448
Low/Moderate	47	29		(33.3)	18	(40)	
High	85	58		(66.7)	27	(60)	
Relapse	132	87			45		0.007
No	98	8		(81.6)	6	(60)	
Yes	34	4		(18.4)	0	(40)	
Ki-67	66	37			29		0.307
Low	34	17		(45.9)	17	(58.6)	
High	32	20		(54.1)	12	(41.4)	

IDC ^1^ = invasive ductal carcinoma; ILC ^2^ = invasive lobular carcinoma; T ^3^ = tumor size; N ^4^ = lymph node metastases.

**Table 2:** Univariate and multivariate Cox analyses in the cohort of 132 TNBC patients.

Parameters		Univariate OS ^1^ Analysis				Multivariate OS Cox Analysis			
		HR ^3^	95% CI ^2^		*p*	HR	95% CI		*p*
			Lower	Upper			Lower	Upper	
T ^4^					0.063				-
	0–1	1.000							
	2–3	2.280	0.957 to 5.433			-	-		
N ^5^					0.014				0.100
	-	1.000				1.000			
	+	2.286	1.180 to 4.429			1.983	0.877 to 4.484		
Grade					0.470				-
	L/M ^6^	1.000							
	High	1.366	0.586 to 3.182			-	-		
Stage					0.049				0.195
	I–II	1.000				1.000			
	III	2.935	1.006 to 8.564			2.174	0.672 to 7.033		
Ki-67					0.864				
	Low	1.000							
	High	1.091	0.402 to 2.962						
pBRD4					<0.001				<0.001
	Low	1.000				1.000			
	High	5.016	2.155 to 11.676			5.342	2.286 to 12.482		

OS ^1^: overall survival; CI ^2^: confidence interval; HR ^3^: Hazard ratio; T ^4^ = tumor size; N ^5^ = lymph node metastases; L/M ^6^: low/moderate.

**Table 3:** Association between pBRD4 expression and PP2A activation status in TNBC patients.

pBRD4	No. Cases	No. Low pBRD4(%)		No. High pBRD4(%)		*p*
pPPP2CA	128	87		41		<0.001
Low	97	85	(97.7)	12	(29.3)	
High	31	2	(2.3)	29	(70.7)	
SET	128	87		41		<0.001
Low	111	87	(100)	24	(58.5)	
High	17	0	(0)	17	(41.5)	

## Data Availability

Data sharing is not applicable for this article.

## Supplementary Materials

The following are available online at https://www.mdpi.com/2072-6694/13/6/1246/s1, Figure S1: Clinical impact of pBRD4 in the cohort of 132 TNBC patients stratified by stage in (A) OS and (B) EFS, Figure S2: Clinical impact of pPPP2CA in the cohort of 132 TNBC patients in (A) OS and (B) EFS, Table S1: Clinical and molecular characteristics in a series of 132 TNBC patients, Table S2: Association between SET and pPPP2CA expression levels in TNBC.
