# Supplementary material for: Expression of Phosphorylated BRD4 Is Markedly Associated with the Activation Status of the PP2A Pathway and Shows a Strong Prognostic Value in Triple Negative Breast Cancer Patients
Source: Cancers (Basel). 2021 Mar 12;13(6):1246. doi: 10.3390/cancers13061246 (PMC7999847; doi:10.3390/cancers13061246)
Supplement: Supplementary file 1 [file cancers-13-01246-s001.pdf]

Supplementary Materials:

Expression of Phosphorylated BRD4 is Markedly Associated with the Activation Status of the PP2A Pathway and Shows a Strong Prognostic Value in Triple Negative Breast Cancer Patients

Marta Sanz-Alvarez, Ion Cristóbal, Melani Luque, Andrea Santos, Sandra Zazo, Juan Madoz-Gúrpide, Cristina Caramés, Cheng-Ming Chiang, Jesús García-Foncillas, Pilar Eroles, Joan Albanell and Federico Rojo

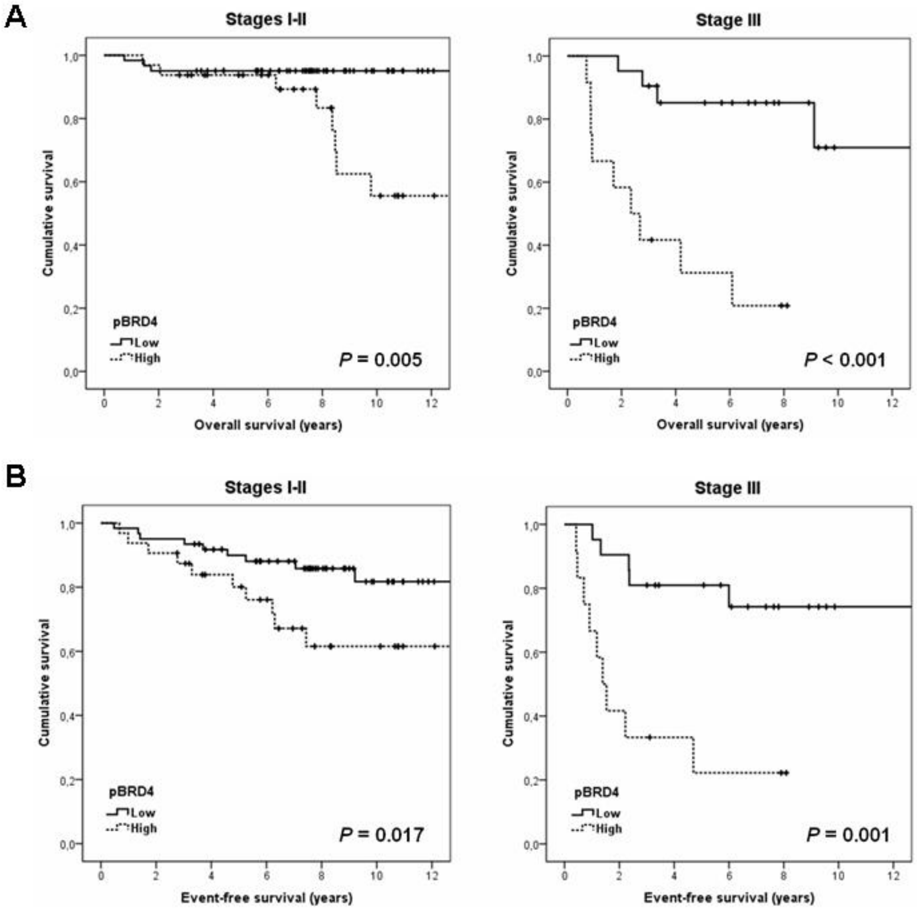

Figure S1. Clinical impact of pBRD4 in the cohort of 132 TNBC patients stratified by stage in (**A**) OS and (**B**) EFS.

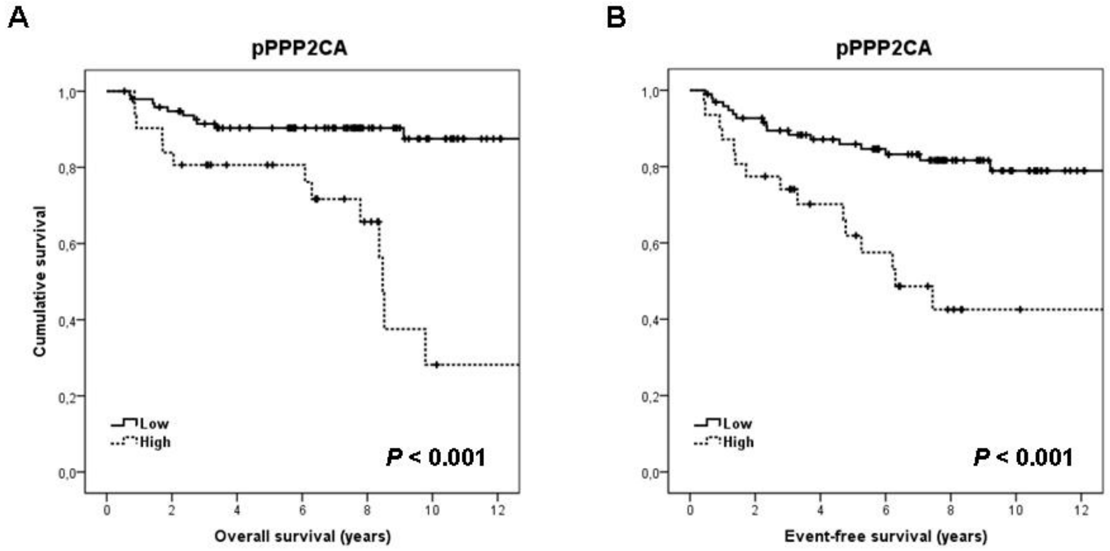

Figure S2. Clinical impact of pPPP2CA in the cohort of 132 TNBC patients in (**A**) OS and (**B**) EFS.

Table S1. Clinical and molecular characteristics in a series of 132 triple negative breast cancer patients.

Variation	Variation	No. (%)	
Age			
Median (range)	57.5 (31–90)		
T			
	1	54	(40.9)
	2	60	(45.5)
	3–4	18	(13.6)
N			
	0	77	(58.3)
	1	33	(25.0)
	2–3	22	(16.7)
Stage			
	I	39	(29.5)
	II	60	(45.5)
	III	33	(25.0)
Grade			
	Low	4	(3.0)
	Moderate	43	(32.6)
	High	85	(64.4)
Morphological type			
	IDC	122	(92.4)
	ILC	9	(6.8)
	Others	1	(0.8)
Hormonal status			
	Premenopausal	36	(27.3)
	Postmenopausal	96	(73.7)
Chemotherapy			
	None	8	(6.1)
	Adjuvant	104	(78.8)
	Neoadjuvant	20	(15.1)
Chemotherapy backbone			
	None	8	(6.1)
	Anthracyclines	49	(37.1)
	Anthracyclines + Taxanes	42	(31.8)
	CMF	33	(25.0)

Relapse			
	No	98	(74.2)
	Yes	34	(25.8)

IDC: invasive ductal carcinoma; ILC: invasive lobular carcinoma.

Table S2. Association between SET and pPPP2CA expression levels in TNBC.

	**No. Cases**	**No. Low SET (%)**		**No. High SET (%)**		***p***
pPPP2CA	128	111		17		**<0.001**
Low	97	92	(82.9)	5	(29.4)	
High	31	19	(17.1)	12	(70.6)	
